# The value of radiography in the follow-up of extremity fractures: a systematic review

**DOI:** 10.1007/s00402-018-3021-y

**Published:** 2018-08-14

**Authors:** P. van Gerven, S. M. Rubinstein, C. Nederpelt, M. F. Termaat, P. Krijnen, M. W. van Tulder, I. B. Schipper

**Affiliations:** 1 Department of Traumasurgery, Leiden University Medical Center, P. O. Box 9600, Postzone K6-R, 2300 RC Leiden, The Netherlands; 2 Department of Health Sciences, Faculty of Science, Amsterdam Public Health Research Institute, VU University, Amsterdam, The Netherlands

**Keywords:** Systematic review, Fractures, Extremity, Radiography, Routine, Added value

## Abstract

**Background:**

The added value of routine radiography in the follow-up of extremity fractures is unclear. The aim of this systematic review was to create an overview of radiography use in extremity fracture care and the consequences of these radiographs for the treatment of patients with these fractures.

**Materials and methods:**

Studies were included if they reported on the use of radiography in the follow-up of extremity fractures and on its influence on treatment strategy, clinical outcome, or complications. A comprehensive search of electronic databases (i.e., PubMed, Embase, and Cochrane) was performed to identify relevant studies. Methodological quality was assessed with the Newcastle–Ottawa scale for cohort studies. Level of evidence was assessed using GRADE. The search, quality appraisal, and data extraction were performed independently by two researchers.

**Results:**

Eleven studies were included. All studies were retrospective cohorts. Of these, only two used a comparative design. Two of the included studies described fractures of both the upper and lower extremities, four studies concerned fractures of the lower extremity only, and five studies focused on fractures of the upper extremity. Pooling of data was not performed because of clinical heterogeneity. Eight studies reported on a change in treatment strategy related to radiography. Percentages ranged from 0 to 2.6%. The overall results indicated that radiographs in the follow-up of extremity fractures seldom alter treatment strategy, that the vast majority of follow-up radiographs are obtained without a clinical indication and that detection of a complication on a radiograph, in the absence of clinical symptoms, is unlikely. All included studies were regarded of a ‘very low’ level using GRADE.

**Conclusions:**

Based on current literature, the added value of routine radiography in the follow-up of extremity fractures seems limited. Results, however, should be interpreted with care, considering that available evidence is of a low level.

**Electronic supplementary material:**

The online version of this article (10.1007/s00402-018-3021-y) contains supplementary material, which is available to authorized users.

## Introduction

Traumatic skeletal fractures are commonly encountered in health care and present a large medical and socio-economic burden [1, 2]. The majority of fractures occur in either the upper or lower extremity. For example, fractures of the wrist, hand, and ankle represent roughly 50% of all skeletal fractures [3]. Due to the aging population, the incidence of extremity fractures is expected to increase in the coming decades [4]. Current national and international protocols recommend frequent outpatient clinic visits at which radiographs of the fractured extremity are obtained. These radiographs can be used to check for (secondary) dislocation, assess bone healing, and provide early detection of complications [5–8]. Other reasons for radiographic imaging include resident education, reassurance of patients, and medicolegal protection [9]. The costs and cost-effectiveness of diagnostic imaging for traumatic skeletal fractures are becoming increasingly important factors in clinical decision making [10]. Recent studies have assessed routine radiography use in patients with distal radius and ankle fractures. These studies suggested that radiographs obtained without a clinical indication do not lead to changes in treatment strategy whilst adding to treatment cost [11–13]. The added value of radiographs for other fractures of the extremities and their consequences for treatment strategies are still unclear. Therefore, the aim of this review was to analyze studies that examine the influence of follow-up radiography for extremity fractures on treatment strategy. Specifically, we focused on whether omission of these more or less routine radiographs is associated with a delayed detection of complications and subsequently a possible deteriorated functional outcome.

## Methods

This systematic review was conducted adhering to the Preferred Reporting Items for Systematic Reviews and Meta-analysis (PRISMA) guidelines [14]. Our methods include a comprehensive search of the literature, independent selection of studies, as well as assessment of the methodologic quality of these studies and extraction of the clinical outcomes by two of the authors.

### Search strategy

A comprehensive literature search was conducted in multiple databases (i.e., Pubmed, Embase, and the Cochrane library) on October 9, 2017. The search strategies were developed with the guidance of a trained medical librarian and included combinations of different terms and synonyms for effectiveness, radiographs, and both upper and lower extremity fractures. In addition, the reference lists of the selected articles were screened for any other relevant studies not identified in the aforementioned electronic search. The search was limited to studies published in the English or Dutch language and was aimed at studies on adult, human subjects. The detailed search strategy is presented in Appendix 1.

The search was repeated on July 10, 2018. In total, 385 additional articles were identified and added to the screening process. No additional relevant studies were found, and thus, none were added to the analysis.

### Criteria for considering studies included in this review

We included studies that described radiographic imaging in the follow-up of fractures of the upper and/or lower extremities. One of the outcome measures had to be either the influence of radiographic imaging on a change in treatment strategy, the association between radiographic imaging and complications (i.e., a lower number of complications detected, or a delayed detection of a complication due to the omission of radiographs) or a possible relation between the omission of radiographs and clinical outcomes (i.e., due to a possible missed complication) such as: range of motion, a functional outcome score (on a validated test/questionnaire), quality of life (using a validated questionnaire), or pain (using a validated instrument). Both randomized controlled trials and observational studies were eligible for inclusion. Case reports and small case series (< 20 subjects) were not included, as well as studies mainly describing patients with pathologic fractures, open fractures (Gustilo grade II/III), severely injured patients (ISS > 16), studies not reporting on the use of radiography in a follow-up setting (but rather in a diagnostic setting), and studies reporting the use of intra-operative control radiographs or their directly post-operative equivalents.

### Selection of studies

After removal of duplicate records, the titles and abstracts of the remaining studies were independently screened by two authors (PvG, CN) using the online systematic review tool “Covidence” (http://www.covidence.org, and Vertitas Health Innovation Ltd) Articles selected based on title and abstract were evaluated fully. If it was unclear whether a study met the inclusion criteria or if no abstract was available, but the title suggested relevance, the full text of the article was assessed for eligibility as well. In the case of a dispute, consensus between the two reviewers was reached by discussion or by consulting an arbiter (SMR), if necessary.

### Assessment of methodological quality

Methodological quality of the included studies was assessed with the Newcastle–Ottawa scale (NOS) by two authors (PvG, CN) independently. In the case of inconsistent results, consensus between the two reviewers was reached by discussion. The Newcastle–Ottawa scale is a frequently used assessment tool for the methodological quality of nonrandomized studies [15]. Separate scales are available for case–control and cohort studies. For this systematic review, we used the scale that evaluates cohort studies, since none of the included studies were randomized or had a case–control design.

The Newcastle–Ottawa scale assesses the methodological quality of studies on eight different criteria distributed over three domains: selection, comparability, and outcome. It is designed to measure the risk of selection bias, information bias, and confounding. Scoring is performed by allocating points when the criteria are met. A total of nine points equals a perfect score. The scale for cohort studies is presented in Appendix 2.

### Data extraction and management

The following study characteristics were extracted: study design, country of origin, fracture location and/or type, number of participants, inclusion and exclusion criteria, participant demographics and study setting, number of (routine) radiographs, outcomes (including: changes in treatment strategy, the number of complications detected on a radiograph, radiographic changes compared to previous imaging or differences in clinical outcome), duration of follow-up, and results. Data extraction was performed by two authors independently (PvG, CN). In the case of a dispute, consensus between the two reviewers was reached by discussion.

### Analysis of results

If the identified studies were clinically homogeneous, a meta-analysis was performed. If the studies were too heterogeneous to pool the data, we performed a descriptive review.

### Assessment of level of evidence

The GRADE method was used to evaluate the overall quality of the evidence and weigh the recommendations [16]. In GRADE, the levels of evidence are stratified high, moderate, low, and very low. Observational studies are primarily labelled ‘low’. A study can gain a ‘level’ if a large (e.g., RR < 0.5) or very large (RR < 0.2) effect was found if there is evidence of a dose–response effect (although this is not applicable to this systematic review) or if plausible residual bias or confounding would only result in study findings being more distinct. On the other hand, a study might drop a ‘level’ if there were limitations in the study design and execution and if there was inconsistency, indirectness, imprecision, or publication bias.

## Results

### Search results

**Fig. 1:**
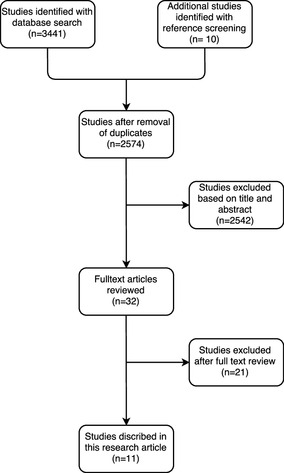
Flowchart of the article selection process

The literature search yielded 2564 unique references. Of these studies, nine were included. Manual screening of reference lists yielded two additional studies. This resulted in 11 unique studies, totaling 4873 participants. The selection process is illustrated in Fig. 1 all studies excluded after full-text review and the reason for exclusion are listed in Appendix 3.

### Study characteristics and overall results

Two of the included studies described fractures of both the upper and lower extremities [17, 18]. Four studies concerned fractures of the lower extremity only [19–22]. The remaining five studies focused on fractures of the upper extremity [23–27]. The extracted characteristics per study are listed in Table 1.All of the included studies used a retrospective cohort design, were conducted in a hospital setting, and evaluated the use of plain radiographs. Two studies compared outcomes between two groups (i.e., one group with a complete set of radiographs as per protocol, and another group, where some radiographs were omitted). Three of the included studies reported on the number of routine radiographs. Ghattas et al. [18] (92.5%), Weil et al. [23] (86%), and Huffaker et al. [25] (94%) all reported that a large majority of follow-up radiographs is not made for a clinical indication. Three studies mainly focused on complications. They concluded that the detection rate of a complication on a radiograph not obtained for a clinical indication was low. Similarly, detection rate of complications was not reduced by the omission of routine radiographs. Mean follow-up length within the studies ranged from 9 days to 64 months. For all studies, this was regarded adequate to evaluate the used outcome measures. The outcome measures that are studied and results of the included studies are reported in Table 2.

**Table 1:** Study characteristics

Author	Years	Country	Fracture location and type	No. of participants	Inclusion/exclusion	Length of follow-up mean (range)	Compared groups	Age mean (range)
de Beaux	1992	Scotland	Elbow joint	31	Incl: patients without a fracture, but with a positive fat pad sign on ED radiographs Excl: no FU radiographs, no-show on 2w visit	2 weeks	–	52 (17–94)
Eastley	2012	UK	Conservatively treated, extra-articular, distal radius fracture	138	Incl: hand therapy, grip strength + ROM. Excl age < 16. Goyrand–Smith fracture, open fracture. NV symptoms, other ER, no initial or follow-up radiographs, instability/pain at follow-up	Until discharge from physiotherapy	Short (1st *X* > 2w, *n* = 77) vs. Long (1st *X* < 2w, *n* = 61)	Short: 63 (17–91) Long: 62 (17–93)
Ghattas	2013	USA	All: pelvis, acetabulum, tibia, ankle, clavicle, elbow, hip, wrist, foot, knee, femur, forearm, humerus, scapula	171 (200 fractures)	Incl: acute fracture age < 18, time to surgery > 2 weeks, surgical fixation with implants, radiograph at 1st post-op visit. Excl: spine and skull fractures	24 days (7–61)	–	58 (18–99)
Huffaker	2014	USA	Distal radius fracture, AO type A	158 (446 radiographs)	Incl: patients with volar locking plate surgery, Excl: open fracture, both bone forearm#, skeletal immature, severely injured patients (ISS > 16)	4.2 months (1.5–48)	–	53.2
Kempegowda	2016	USA	Healed intertrochanteric fracture	465	Incl: clinical and radiological consolidation, FU > 1Y Excl: age < 60, pathological fracture, periprosthetic fracture, sec. dislocation, non-union	81.2 weeks (52–368)	–	77 (60–98)
McDonald	2014	USA	Operatively treated ankle fracture	1411	Incl :surgical fixation Excl: open fracture, incomplete charts, no radiography between T + 7 and T + 120 days	Until discharge from clinic	Early (*X* w 1–3 *n* = 889) vs. Late (*X* > 3w, *n* = 522)	Early 36 (21–52) Late 40 (25–55)
Ovaska	2016	Finland	Operatively treated ankle fracture	878	Incl: age 16 + ORIF of the fracture	64 months	–	48 (16–91)
Robertson	2000	Scotland	Isolated, closed tibial shaft fracture	53 (343 radiographs)	Incl: treated with intramedullary nailing	No statement. time to union: 24 weeks (10–73)	–	31 (14–86)
Schuld	2016	USA	Non-displaced fracture of hand, wrist, ankle or foot	265 (27 post-splinting X, 179 repeat X at FU)	Incl: non-dislocated fracture, plaster immobilization. Excl: brace immobilization	9 days (1–135)	–	34 (1–91)
Stone	2015	USA	Operatively treated distal radius fracture	261 (268 fractures)	Excl: skeletal immaturity, absent 2-week radiograph, less than 3 sets of radiographs	12 weeks	–	29 (14–90)
Weil	2017	NL	Both operatively and conservatively treated distal radius fractures	1042	Incl: age > 18 Excl: absence of FU data, pathologic fracture, open fracture, >1 simultaneous fracture of the extremities	No statement	–	58,5 (SD:19.6)

Characteristics of the studies included in the systematic review

*UK* United Kingdom, *USA* United States of America, *NL* Netherlands, *AO* Arbeitsgemeinschaft für Osteosynthesefragen^1^, *ROM* range of motion, *FU* follow-up, *ISS* injury severity score, *ER* emergency room, *ORIF* open reduction, internal fixation, *SD* standard deviation, *T + x* x days following trauma or operative fixation, *X* radiograph

1. *AO foundation*. Available from: http://www.aofoundation.org

**Table 2:** Study outcomes

Author	Relevant measured outcome(s)	Changes in management	Results
de Beaux	Change in treatment strategy	0/31 (0%)	6% fractures observed (2 patients), no changes treatment strategy
Eastley	Grip strength, ROM, conversion to operative care	0/61 (0%)	Grip strength/ROM: no difference. no conversion to operative care based on late radiographs
Ghattas	No. of radiographs per patient, changes from normal post-operative management	3/200 (1.5%)	3/200 changes from normal post-operative management
Huffaker	% Clinical findings (changes from expected normal follow-up), % radiographic findings(hardware or fracture complications), re-intervention, complications	–	0% radiographic complications
Kempegowda	Changes on radiographs obtained after radiological healing had been established. no. of radiographs and clinic visits, complications, costs	–	No. of clinic visits: 2.8, no. of Radiographs: 2.6. 98% no changes, 0.7% AVN 0.7% osteoarthritis 0.7% heterotopic ossification
McDonald	Complications	–	Complications: early: 62/889 (7.0%) late 31/522(5.9%) *P* = 0.45
Ovaska	Change in treatment strategy	3/878 (0.3%)	3/878 changes in treatment strategy based merely on radiographs (0.3%)
Robertson	Change in treatment strategy	9/343 (2,6%)	9/343 (2,6%) of follow-up radiographs --> change in treatment strategy
Schuld	Dislocation on post-splinting radiographs. secondary displacement on repeat radiographs, change in treatment strategy	0/27 (0%)	No change in treatment strategy based on post-splinting radiographs. 7.8% sec. dislocation. No change in treatment strategy based on repeat radiographs
Stone	change from normal post-operative management, unplanned re-intervention	3/261 (1.1%)	1% unexpected changes in post-operative management (3 Patients) (secondary dislocation/hardware failure) --> re-intervention (all after new trauma)
Weil	Change in treatment strategy	11/720 (1.5%)	Change in treatment strategy: 22/841 radiographs (2.6%). Changes based on routine radiographs: 11/720 (1.5%). 9/11 (1.2%) prolonged cast immobilization, 2/11 (0.2%) conversion to surgery

Measured outcomes and results of included studies

*ROM* range of motion

The included articles were clinically too heterogeneous for pooling of data to be meaningful. We therefore chose to describe the results of the individual studies.

### Methodological quality

On the Newcastle–Ottawa scale, the included studies earned a total number of three-to-six points, out of a maximum of nine. For the selection domain, the maximum achieved score was three points out of a maximum of four. Since we identified only retrospective studies, none of the studies got a point for item four ‘demonstration that the outcome of interest was not present at the start of the study’. Schuld et al. [17], McDonald et al. [19], and Eastley et al. [26] scored three points in the selection domain. All other studies, with the exception of Robertson et al. [22], scored two points, since there was no non-exposed cohort. None of the studies fulfilled the criteria for comparability, given that none controlled for baseline factors. Six studies (i.e., McDonald et al. [19], Ovaska et al. [20], Kempegowda et al. [21], Weil et al. [23], Stone et al. [24], and Huffaker et al. [25]) scored the maximum number of three points for the outcome domain. All other studies scored two points, mainly because no statement was made on the adequacy of follow-up. An overview of the scores is presented in Table 3.

**Table 3:** Methodological quality

Author	Selection (max 4 ★)	Comparability (max 2★)	Outcome (max 3★)
De Beaux	★★	–	★★
Eastley	★★★	–	★★
Ghattas	★★	–	★★
Huffaker	★★	–	★★★
Kempegowda	★★	–	★★★
McDonald	★★★	–	★★★
Ovaska	★★	–	★★★
Robertson	★	–	★★
Schuld	★★★	–	★★
Stone	★★	–	★★★
Weil	★★	–	★★★

Scores per category on the Newcastle–Ottawa scale

### Results on outcome measures from individual studies

#### Fractures of both the upper and lower extremities

Two studies found no changes in treatment strategy for post-splinting and post-operative radiographs of both the upper and lower extremities.

Schuld et al. [17] (NOS 5/9) examined the effect of imaging on the treatment of 265 non-displaced fractures of the hand, wrist, ankle, or foot. They examined the number of dislocations during the splinting procedure on post-splinting X-rays (*n* = 27) and the number of secondary dislocations in patients with follow-up radiographs obtained at the outpatient clinic (*n* = 179). No changes in management based on post-splinting radiographs were identified. Secondary dislocation was observed in of 7.8% of participants (*n* = 14). Treatment strategy was unaltered in all of these patients. Based on these findings, post-splinting radiographs were labelled “likely unnecessary”, and the authors stated that repeat imaging in this patient group should be discouraged.

Ghattas et al. [18] (NOS 4/9) assessed the influence of radiographs on the treatment strategy of extremity fractures that were treated with surgical fixation in a retrospective, 2-year cohort. In total, 200 fractures in 171 patients were included. All changes to normal post-operative management (i.e., all procedures or interventions not typically used in the aftercare of that specific fracture) at the initial outpatient clinic visit were identified. Over a mean follow-up period of 24 days (range 7–61 days), 3 out of 200 fractures had a change in treatment strategy. All three changes were based on clinical symptoms, rather than on the radiographs. The authors concluded that radiographs at the initial post-operative outpatient clinic visit do not alter treatment strategy, but do pose a financial burden.

#### Fractures of the lower extremity

Four studies showed that radiographs of the lower extremity do not change treatment strategy, do not have an impact on complications, and should not be obtained if there are no clinical signs of a complication.

McDonald et al. [19] (NOS 6/9) studied the number of complications in relation to the timing of the first post-operative radiograph in a retrospective cohort of 1411 operatively treated ankle fractures. They divided this cohort in two groups. The first group had their initial follow-up radiograph taken in the first 3 weeks following surgery; the second received their initial follow-up radiograph more than 3 weeks after the intervention. They observed 62 complications in 889 patients with ‘early’ radiographs (7.0%), and 31 complications in 522 patients with radiographs solely obtained more than 3 weeks after surgery (5.9%). This difference was not statistically significant. The researchers concluded that obtaining early routine radiographs (i.e., in the first 3 weeks following surgery) for all patients with an ankle fracture is of questionable benefit.

Ovaska et al. [20] (NOS 5/9) evaluated the number of changes in treatment strategy based on radiographs obtained at the first scheduled outpatient clinic visit in a retrospective cohort of 878 patients with an operatively treated ankle fracture. In three out of 878 patients (0.3%), a change in treatment strategy was observed solely based on a routine radiograph. All of these changes were adjustments in weight bearing regimen, either after an initially undiagnosed medial malleolus fracture, or after subtle secondary dislocation. The authors concluded that routine radiographs should probably not be obtained at the first outpatient clinic visit if no clinical signs of a complication are present.

Kempegowda et al. [21] (NOS 5/9) assessed a cohort of 465 patients with healed intertrochanteric fractures with a mean follow-up period of 81 weeks. The main outcome measure was a radiological change on radiographs obtained after clinical and radiological union had already been demonstrated earlier on. On average, patients had 2.8 outpatient clinic visits, and 2.6 radiographs after union had been confirmed. Of these radiographs, 98% did not reveal changes when compared to previous imaging. Three images (0.7%) showed signs of avascular necrosis of the femoral head, three showed osteoarthritis of the hip, and three revealed heterotopic ossification. The authors concluded that there is a negligible role for radiographs and clinic visits when evidence of clinical and radiographic healing with acceptable alignment of an intertrochanteric fracture is available.

Robertson et al. [22] (NOS 3/9) retrospectively evaluated 53 patients with an isolated tibial shaft fracture that were treated with an intramedullary nail. Out of 343 radiographs obtained during follow-up, nine (3%) directly led to a change in clinical management. In two patients, radiographs showed union, and the nail was removed. The remaining seven patients showed signs of delayed union, which gave rise to nail exchange procedures. The authors concluded that serial radiographs are not justified, and that radiographs prior to 10-week follow-up should only be obtained when there is a clinical suspicion of a complication.

#### Fractures of the upper extremity

Five studies showed that follow-up radiographs of the upper extremity seldom influenced treatment strategy, should only be obtained on a clinical indication and that routine radiography can probably be omitted.

Weil et al. [23] (NOS 5/9) evaluated the use of routine radiographs, and the changes in treatment strategy based on these radiographs, taken after more than 3 weeks of follow-up in a multi-center cohort of 1042 patients with a distal radius fracture. A radiograph was labelled routine if no clinical indication for obtaining it was registered in the medical records. Of the 720 radiographs that complied with these requirements, 11 (1.5%) led to a change in treatment strategy. In nine instances, cast immobilization was prolonged, and in two instances, the patient was converted to operative treatment. The conclusion of the authors was that routine radiographs after the initial 3-week follow-up period seldom influence clinical decision making.

Stone et al. [24] (NOS 5/9) studied radiographs taken 2 weeks after open reduction and internal fixation of distal radius fractures in a retrospective cohort of 261 patients with 268 fractures. They evaluated the number of changes in treatment strategy as well as the number of re-interventions. At 2-week follow-up, three changes in management were recorded (1.1%). All of these cases involved patients with a loss of reduction or hardware failure after a consecutive trauma to the injured wrist. The authors concluded that for low-energetic, non-comminuted fractures, routine radiographs at 2 weeks could be omitted.

Huffaker et al. [25] (NOS 5/9) evaluated the value of routine post-operative radiographs in AO type A [28] distal radius fractures treated with volar locking plates. They identified 446 post-operative radiographs in a cohort of 158 patients. During follow-up (mean 4.2 months), none of the radiographs showed non-union, loss of fixation or a change in alignment. For patients presenting with symptoms (such as neuropathy, signs of infection, pain, or crepitation), radiography was not associated with a higher likelihood of operative intervention. The authors concluded that radiographs, apart from the primary direct post-operative radiograph, should only be obtained for a clinical indication.

Eastley et al. [26] (NOS 5/9) assessed 137 patients with extra-articular distal radius fractures that were treated non-operatively. They investigated whether grip strength, clinical deformity, and range of motion were associated with obtaining radiographs after more than 2 weeks of follow-up. The cohort was divided into two groups. One that had radiographs taken only in the first 2 weeks (‘early’ *n* = 77), and another group that had follow-up radiographs beyond this term as well (‘late’ *n* = 61). No significant differences in grip strength, mean flexion, dorsiflexion, radial deviation, and ulnar deviation were found. There was no conversion to operative care based on late radiographs. The authors concluded that omission of late radiographs in this patient category may have no adverse effects on clinical outcome whilst providing financial benefits.

De Beaux et al. [27] (NOS 4/9) evaluated a retrospective cohort of 45 patients with a suspected fracture of the elbow region (depicted by a positive fat pad sign, but the absence of a fracture line on the initial emergency room radiographs). The main research question was if repeat radiography after 2-week altered treatment strategy. At the 2-week follow-up moment, 11 patients failed to attend and 3 had no repeat radiographs made. Of the remaining 31 patients, 29 had normal radiographs, and 2 patients were diagnosed with a non-displaced fracture of the radial head. No changes were made to the treatment of any participant. The authors concluded that routine follow-up radiography is unnecessary in this patient category.

### Level of evidence

All of the included studies are observational, and therefore, the initial level of evidence should be considered ‘low’. Since the studies are retrospective in nature, the risk of bias was regarded high. As a result, the level of evidence was downgraded to ‘very low’ for all included studies.

## Discussion

In total, we identified 11 retrospective studies that examined the possible relation between radiographic imaging and treatment strategy. Several studies also described the influence of the omission of radiographs on functional outcome or detection of complications. Unfortunately, these studies were clinically so diverse that it was not possible to pool the data. Based upon the descriptive analysis, it appears that all studies come to essentially the same conclusion. They all suggest that omitting some, or even all, follow-up radiographs of extremity fractures does not have important clinical consequences, such as changes in treatment strategy, a deterioration of clinical outcomes, or missed complications. From the studies we included in this systematic review, no distinction could be made between different fracture locations or fracture types. However, all conclusions were based upon retrospective studies, introducing a high risk of bias and confounding. The level of evidence was low, indicating that these results should be interpreted with caution. We did not identify any prospective studies. As a result, studies included in this review should be regarded as the best available evidence at present.

For other indications, such as low back pain [29], knee osteoarthritis [30], or following paediatric spinal surgery [31] the added value of routine radiographs are being questioned as well. Apparently, for other indications than extremity fractures, radiographs are also obtained routinely and without great impact on treatment strategies. In addition, for direct post-operative check radiographs of, for instance, hip fractures, multiple retrospective studies exist that debate their usefulness or discourage their use [32–35]. A randomized study investigating the usefulness of direct post-operative control radiographs for operatively treated wrist and ankle fractures is currently being conducted by Oehme et al. [36]. Routine radiographs might resemble low-value care, and omitting them might lead to increased efficiency for the health care system. The American College of Foot and Ankle Surgeons released a consensus statement discouraging the use of routine radiographs to monitor fracture, osteotomy, and arthrodesis healing without a clinical indication in the foot and ankle [37]. However, to date, prospective evidence to support this claim is lacking.

In all studies included in this review, the number of changes in treatment strategy based on radiography was low. As depicted in Table 3, it ranged from 0 to 2.6%. The number of complications detected on a routine radiograph, in the absence of clinical symptoms, was similarly low. Both patients and physicians tend to ascertain value to radiographic confirmation of a favourable recovery. However, this review suggests that findings on a routine radiograph that require a change in treatment strategy, in the absence of clinical symptoms, are rare. The presence of clinical symptoms could be a good predictor of an unfavorable outcome, and might justify the use of radiography to rule out a complication. In the randomized controlled trial we are currently conducting [38], reasons to obtain radiographs include: a pain score higher than 6 on a 1–10 Visual Analog Scale, a loss in range of motion, neurovascular symptoms, or a successive trauma to the injured limb. It is clear from our overview that interest in this topic is growing. All but two studies were published in the last 6 years, and quality and precision of the studies improved over time. For example, the older two studies contributed just 2% to the total number of participants and scored poorly on the Newcastle–Ottawa scale (three and four points out of nine, respectively). The more recent studies included more participants and, on average, scored higher on the Newcastle–Ottawa scale.

### Limitations and strengths

All studies included in this review had a retrospective design and several other limitations in their study design on the Newcastle–Ottawa scale. All studies but two had a non-comparative design, and no statistical testing of outcomes was performed. The risk of bias was high, confounding was likely, and the external validity was limited. This resulted in a ‘very low’ level of evidence according to GRADE.

Conclusions in systematic reviews are dependent on the quality and design of studies included. The fact that only retrospective studies were identified and the level of evidence was very low hinders us in making strong recommendations. A second potential limitation was the tool used for assessment of the methodological quality of the included studies. The Newcastle–Ottawa scale is best suited for comparative and prospective non-randomized studies; therefore, this tool might not deliver the best assessment of risk of bias in the current setup. Finally, we limited our search to English and Dutch; therefore, language bias may affect our conclusions. However, no studies in Dutch were identified by the search strategy, and manual screening of the reference lists of included studies did not yield any references in a language other than English. Consequentially, the chance that language bias played a substantial role in the selection process of the systematic review was deemed low.

A strength of this study is presented by the fact that the percentage of included studies was very low (0.4%). This indicates that our initial search was broad, and as a result, the risk that important publications were missed was low.

## Conclusion

The added value of routine radiography in extremity fractures appears limited, whilst making these radiographs involves effort and cost. Although this conclusion is based upon results of retrospective studies with all concomitant limitations, some reservation in use of follow-up radiographs for extremity fractures seems justified. We urge physicians to be reticent in ordering follow-up radiographs of lower and upper extremity fractures in the absence of a clear clinical indication. Future research in this topic should focus on the conception of prospective randomized studies. These studies should evaluate the impact of routine radiographic imaging on treatment strategy and treatment outcomes of patients with extremity fractures. Conducting such a trial seems feasible and might provide a more solid substantiation of our conclusion.

## Electronic supplementary material

Below is the link to the electronic supplementary material.

Supplementary material 1: Appendix 1: Search strategy (DOCX 28 KB)

Supplementary material 2: Appendix 2: Newcastle-Ottawa Scale (PDF 21 KB)

Supplementary material 3: Appendix 3: Excluded articles based on fulltext (DOCX 15 KB)
