# Supplementary material for: The value of radiography in the follow-up of extremity fractures: a systematic review
Source: Arch Orthop Trauma Surg. 2018 Aug 14;138(12):1659–69. doi: 10.1007/s00402-018-3021-y (PMC6224023; doi:10.1007/s00402-018-3021-y)
Supplement: Supplementary file 1 — Supplementary material 1: Appendix 1: Search strategy (DOCX 28 KB) [file 402_2018_3021_MOESM1_ESM.docx]

**PubMed/Cochrane**

**#1 Reduce frequency Radiography**

"Radiography"[Mesh] OR "Radiation"[Mesh] OR Diagnostic X-Ray*[tiab] OR Roentgenograph*[tiab] OR Roentgenogram*[tiab] OR X-Ray Radiolog*[tiab] OR reduced imaging[tiab] OR radiograph*[tiab] OR "diagnostic imaging"[Subheading] OR radiation[tiab] OR (Imaging[tiab] AND (protocol*[tiab] OR "standards"[Subheading] OR standards[tiab] OR guideline*[tiab] OR criteria*[tiab] OR practice*)) **AND** ("Diagnostic Tests, Routine"[Mesh] OR "Unnecessary Procedures/economics"[Mesh] OR "Unnecessary Procedures/epidemiology"[Mesh] OR Reducing[tiab] OR omitt*[tiab] OR omission[tiab] OR frequenc*[tiab] OR decreas*[tiab] OR lessen[tiab] OR restrict*[tiab] OR cut down[tiab] OR routine*[tiab])

**#2a bones of upperextremity**

(("Upper Extremity"[Mesh] OR Upper Extremit*[tiab] OR Membrum superius[tiab] OR Upper Limb*[tiab] OR "Bones of Upper Extremity"[Mesh] OR arm[tiab] OR arms[tiab] OR brachium*[tiab] OR shoulder[tiab] OR clavic*[tiab] OR collar bone*[tiab] OR scapula*[tiab] OR shoulder blade*[tiab] OR acromion*[tiab] OR coracoid*[tiab] OR glenoid[tiab] OR humerus[tiab] OR humeral[tiab] OR Tuberc*[tiab] OR tuberosity*[tiab] OR trochlea*[tiab] OR epicondy*[tiab] OR condy*[tiab] OR elbow*[tiab] OR ulna*[tiab] OR olecran*[tiab] OR radius[tiab] OR radial[tiab] OR coranoid*[tiab] OR forearm*[tiab] OR Antebrachi*[tiab] OR wrist*[tiab] OR hand[tiab] OR hands[tiab] OR finger*[tiab] OR thumb*[tiab] OR carpus[tiab] OR carpal[tiab] OR scaphoid*[tiab] OR navicular*[tiab] OR triquetra*[tiab] OR Metacarp*[tiab] OR phalanges[tiab] OR phalanx[tiab]) AND ("Fractures, Bone"[Mesh:NoExp] OR "Fracture Healing"[Mesh] OR fracture*[tiab] OR broken bone*[tiab]))

**Specific types of upper extremity fractures**

"Shoulder Fractures"[Mesh] OR "Humeral Fractures"[Mesh] OR "Ulna Fractures"[Mesh]  OR Monteggia*[tiab] OR galeazz*[tiab] OR "essex lopresti"[tiab] OR "Radius Fractures"[Mesh] OR Colles*[tiab] OR boxers fracture*[tiab] OR boxer’s fracture*[tiab] OR bankart[tiab] OR hill-sachs[tiab] OR Bennett*[tiab] OR Rolando*[tiab] OR smith’s fracture*[tiab] OR Goyrand-Smith's[tiab]

**#2b bones of lower extremity**

(("Lower Extremity"[Mesh] OR Lower Extremit*[tiab] OR lower limb[tiab] OR membrum inferius[tiab] OR "Bones of Lower Extremity"[Mesh] OR leg bone*[tiab] OR hip fracture*[tiab] OR fracture of the hip[tiab] OR fractures of the hip[tiab] OR Femur*[tiab] OR femoral*[tiab] OR trochanter*[tiab] OR intertrochanter*[tiab] OR subtrochanter*[tiab] OR patella*[tiab] OR knee[tiab] OR knees[tiab] OR kneecap*[tiab] OR tibia*[tiab] OR fibula*[tiab] OR foot bone*[tiab] OR feet bone*[tiab] OR Tarsal*[tiab] OR ankle*[tiab] OR cuneiform*[tiab] OR cuboid*[tiab] OR calcaneus*[tiab] OR heel bone*[tiab] OR metatarsal*[tiab] OR toe bone*[tiab] OR toes bone*[tiab] OR hallux*[tiab] OR hallic*[tiab] OR malleol*[tiab] OR trimall*[tiab] OR bimall*[tiab]) AND ("Fractures, Bone"[Mesh:NoExp] OR "Fracture Healing"[Mesh] OR fracture*[tiab] OR broken bone*[tiab]))

**Specific types of lower extremity fractures**

"Femoral Fractures"[Mesh] OR "Tibial Fractures"[Mesh] OR "Ankle Fractures"[Mesh] OR maisonneuve*[tiab] OR lisfranc*[tiab] OR segond*[tiab] OR tillaux*[tiab]

**#3 QoL/ outcome measurements**

"Health Status"[mesh] OR "Quality of Life"[mesh] OR "Treatment Outcome"[mesh] OR "Outcome Assessment (Health Care)"[Mesh] OR "Recovery of Function"[Mesh] OR "Clinical Decision-Making"[Mesh] OR clinical indicat*[tiab] OR clinical impact*[tiab] OR treatment strategy[tiab] OR therapeutic polic*[tiab] OR patient management[tiab] OR management policy[tiab] OR clinical management[tiab] OR recovery[tiab] OR "Health Status"[tiab] OR "Quality of Life"[tiab] OR clinical Outcome*[tiab] OR value[tiab] OR "clinical decision making"[tiab] OR "Quality-Adjusted Life Years"[Mesh] OR (("Life years"[tiab]) AND ("Quality adjusted"[tiab] OR adjusted[tiab] OR Gained [tiab])) OR "QUALY"[tiab] OR "LYG" [tiab] OR "Quality adjusted"[tiab] OR ((change*[tiab] OR changing[tiab]) AND management*[tiab])

**#4 Adults only**

NOT (("Adolescent"[Mesh] OR "Child"[Mesh] OR "Infant"[Mesh] OR adolescen*[tiab] OR child*[tiab] OR schoolchild*[tiab] OR infant*[tiab] OR girl*[tiab] OR boy*[tiab] OR teen[tiab] OR teens[tiab] OR teenager*[tiab] OR youth*[tiab] OR pediatr*[tiab] OR paediatr*[tiab] OR puber*[tiab]) NOT ("Adult"[Mesh] OR adult*[tiab] OR man[tiab] OR men[tiab] OR woman[tiab] OR women[tiab]))

**#5 Publication types/ humans**

NOT ("addresses"[Publication Type] OR "biography"[Publication Type] OR "Case Reports" [Publication Type] OR "comment"[Publication Type] OR "directory"[Publication Type] OR "editorial"[Publication Type] OR "festschrift"[Publication Type] OR "interview"[Publication Type] OR "lectures"[Publication Type] OR "legal cases"[Publication Type] OR "legislation"[Publication Type] OR "letter"[Publication Type] OR "news"[Publication Type] OR "newspaper article"[Publication Type] OR "patient education handout"[Publication Type] OR "popular works"[Publication Type] OR "congresses"[Publication Type] OR "consensus development conference"[Publication Type] OR "consensus development conference, nih"[Publication Type] OR "practice guideline"[Publication Type]) NOT ("animals"[MeSH Terms] NOT "humans"[MeSH Terms])

**EMBASE.com**

**#1 Reduce frequency Radiography**

'radiography'/exp OR 'radiation'/exp OR Diagnostic X-Ray*:ti,ab OR Roentgenograph*:ti,ab OR Roentgenogram*:ti,ab OR X-Ray Radiolog*:ti,ab OR 'reduced imaging':ti,ab OR radiograph*:ti,ab OR 'diagnostic imaging':ti,ab OR radiation:ti,ab OR (Imaging:ti,ab AND (protocol*:ti,ab OR standards:ti,ab OR guideline*:ti,ab OR criteria*:ti,ab OR practice*)) AND ('diagnostic test'/exp OR 'unnecessary procedure'/exp OR Reducing:ti,ab OR omitt*:ti,ab OR omission:ti,ab OR frequenc*:ti,ab OR decreas*:ti,ab OR lessen:ti,ab OR restrict*:ti,ab OR 'cut down':ti,ab OR routine*:ti,ab)

**#2a bones of upperextremity**

(('upper limb'/exp OR 'Upper Extremit*':ti,ab OR 'Membrum superius':ti,ab OR 'Upper Limb*':ti,ab OR 'bones of the arm and hand'/exp OR arm:ti,ab OR arms:ti,ab OR brachium*:ti,ab OR shoulder:ti,ab OR clavic*:ti,ab OR 'collar bone*':ti,ab OR scapula*:ti,ab OR 'shoulder blade*':ti,ab OR acromion*:ti,ab OR coracoid*:ti,ab OR glenoid:ti,ab OR humerus:ti,ab OR humeral:ti,ab OR Tuberc*:ti,ab OR tuberosity*:ti,ab OR trochlea*:ti,ab OR epicondy*:ti,ab OR condy*:ti,ab OR elbow*:ti,ab OR ulna*:ti,ab OR olecran*:ti,ab OR radius:ti,ab OR radial:ti,ab OR coranoid*:ti,ab OR forearm*:ti,ab OR Antebrachi*:ti,ab OR wrist*:ti,ab OR hand:ti,ab OR hands:ti,ab OR finger*:ti,ab OR thumb*:ti,ab OR carpus:ti,ab OR carpal:ti,ab OR scaphoid*:ti,ab OR navicular*:ti,ab OR triquetra*:ti,ab OR Metacarp*:ti,ab OR phalanges:ti,ab OR phalanx:ti,ab) AND ('fracture'/de OR 'fracture healing'/exp OR fracture*:ti,ab OR 'broken bone*':ti,ab))

**Specific types of upper extremity fractures**

'shoulder fracture'/exp OR 'humerus fracture'/exp OR 'ulna fracture'/exp OR Monteggia*:ti,ab OR galeazz*:ti,ab OR 'essex lopresti':ti,ab OR 'radius fracture'/exp OR Colles*:ti,ab OR 'boxers fracture*':ti,ab OR 'boxers fracture*':ti,ab OR bankart:ti,ab OR  'hill-sachs':ti,ab OR Bennett*:ti,ab OR Rolando*:ti,ab OR 'smiths fracture*':ti,ab OR 'Goyrand-Smith*':ti,ab

**#2b bones of lower extremity**

(('lower limb'/exp OR 'Lower Extremit*':ti,ab OR 'lower limb':ti,ab OR 'membrum inferius':ti,ab OR 'bones of the leg and foot'/exp OR 'leg bone*':ti,ab OR 'hip fracture*':ti,ab OR 'fracture of the hip':ti,ab OR 'fractures of the hip':ti,ab OR Femur*:ti,ab OR femoral*:ti,ab OR trochanter*:ti,ab OR intertrochanter*:ti,ab OR subtrochanter*:ti,ab OR patella*:ti,ab OR knee:ti,ab OR knees:ti,ab OR kneecap*:ti,ab OR tibia*:ti,ab OR fibula*:ti,ab OR 'foot bone*':ti,ab OR 'feet bone*':ti,ab OR Tarsal*:ti,ab OR ankle*:ti,ab OR cuneiform*:ti,ab OR cuboid*:ti,ab OR calcaneus*:ti,ab OR heel bone*:ti,ab OR metatarsal*:ti,ab OR 'toe bone*':ti,ab OR 'toes bone*':ti,ab OR hallux*:ti,ab OR hallic*:ti,ab OR malleol*:ti,ab OR trimall*:ti,ab OR bimall*:ti,ab) AND ('fracture'/de OR 'fracture healing'/exp OR fracture*:ti,ab OR 'broken bone*':ti,ab))

**Specific types of lower extremity fractures**

'femur fracture'/exp OR 'tibia fracture'/exp OR 'ankle fracture'/exp OR maisonneuve*:ti,ab OR lisfranc*:ti,ab OR segond*:ti,ab OR tillaux*:ti,ab

**#3 QoL/ outcome measurements**

'health status'/exp OR 'quality of life'/exp OR 'treatment outcome'/exp OR 'outcome assessment'/exp OR 'convalescence'/exp OR 'clinical decision making'/exp OR 'clinical indicat*':ti,ab OR 'clinical impact*':ti,ab OR 'treatment strategy':ti,ab OR 'therapeutic polic*':ti,ab OR 'patient management':ti,ab OR 'management policy':ti,ab OR 'clinical management':ti,ab OR recovery:ti,ab OR 'Health Status':ti,ab OR 'Quality of Life':ti,ab OR 'clinical Outcome*':ti,ab OR value:ti,ab OR 'clinical decision making':ti,ab OR 'quality adjusted life year'/exp OR (('Life years':ti,ab) AND ('Quality adjusted':ti,ab OR adjusted:ti,ab OR gained:ti,ab)) OR 'QUALY':ti,ab OR 'LYG':ti,ab OR 'Quality adjusted':ti,ab OR ((change*:ti,ab OR changing:ti,ab) AND management*:ti,ab)

**#4 Adults only**

NOT (('juvenile'/exp OR 'embryo'/exp OR 'fetus'/exp OR adolescen*:ti,ab OR child*:ti,ab OR schoolchild*:ti,ab OR infant*:ti,ab OR girl*:ti,ab OR boy*:ti,ab OR teen:ti,ab OR teens:ti,ab OR teenager*:ti,ab OR youth*:ti,ab OR pediatr*:ti,ab OR paediatr*:ti,ab OR puber*:ti,ab) NOT ('adult'/exp OR adult*:ti,ab OR man:ti,ab OR men:ti,ab OR woman:ti,ab OR women:ti,ab))

**#5 Publication types/ humans**

PT use EMBASE filters

NOT ('animal'/exp] NOT 'human'/exp)
