# Supplementary material for: The value of radiography in the follow-up of extremity fractures: a systematic review
Source: Arch Orthop Trauma Surg. 2018 Aug 14;138(12):1659–69. doi: 10.1007/s00402-018-3021-y (PMC6224023; doi:10.1007/s00402-018-3021-y)
Supplement: Supplementary file 3 — Supplementary material 3: Appendix 3: Excluded articles based on fulltext (DOCX 15 KB) [file 402_2018_3021_MOESM3_ESM.docx]

| **Author** | **Year** | **Journal** | **Reason for exclusion** |
| --- | --- | --- | --- |
| Archdeacon | 2015 | J Orthop Trauma | Study describing direct postoperative 'check radiography' |
| Bessette | 2017 | J Arthroplasty | Study describing patients with an artroplasty, not a fracture |
| Bhattacharyya | 2017 | Injury | Study describing a reduction in outpatient clinic visit, not radiography |
| Chakravarthy | 2007 | Int J Clin Pract | Study describing direct postoperative 'check radiography' |
| Chaudhry | 2012 | J Bone Joint Surg Am | Study describing direct postprocedural 'check radiography' |
| Ferguson | 2015 | Injury | Study describing a reduction in outpatient clinic visit, not radiography |
| Harish | 1999 | Injury | Study describing direct postprocedural 'check radiography' |
| Jain | 2008 | Ann R Coll Surg Engl | Study not reporting on any of the required outcome measures |
| Johnson | 2016 | Plast Reconstr Surg | Study describing direct postoperative 'check radiography' |
| Kurup | 2008 | Eur J Orthop Surg Traumatol | Study describing direct postoperative 'check radiography' |
| Michelson | 1995 | J Trauma | Study not reporting on any of the required outcome measures |
| Miniaci-Coxhead | 2015 | Foot Ankle Int | Study describing direct postoperative 'check radiography' |
| Mohanti | 2000 | J R Coll Surg Edinb | Study describing direct postoperative 'check radiography' |
| Moody | 2016 | J Orthop Trauma | Study not reporting on any of the required outcome measures |
| Morewood | 1987 | Br. Med J (Clin Res Ed) | Study with a large percentage (26%) of pediatric patients |
| O'Shea | 2006 | J Orthop Surg (Hong Kong) | Study describing direct postoperative 'check radiography' |
| Pannell | 2016 | Hand (N Y) | Study without "clinical" cases, solely imaging. |
| Quinton | 1987 | J Bone Joint Surg Br | Study describing patients without a fracture. |
| Stott | 2017 | J Orthop Trauma | Study describing direct postoperative 'check radiography' |
| Welsh | 1987 | Br Med J (Clin Res Ed) | Correspondence with comment on a research article (Morewood 1987) |
| Westerterp | 2013 | Eur J Trauma Emerg Surg | Study describing direct postoperative 'check radiography' |
